# Eating Disorders: An Analysis of Self-Induced Vomiting, Binge Eating, and Oral Hygiene Behavior

**DOI:** 10.1155/2022/6210372

**Published:** 2022-04-23

**Authors:** Ann-Katrin Johansson, Tonje Mjanger Øvretvedt, Karen Knudsen Reinholtsen, Anders Johansson

**Affiliations:** ^1^Department of Clinical Dentistry-Cariology, Faculty of Medicine, University of Bergen, Bergen, Norway; ^2^Department of Clinical Dentistry-Prosthodontics, Faculty of Medicine, University of Bergen, Bergen, Norway

## Abstract

**Background:**

Self-induced vomiting (SIV) is often present in patients with eating disorders (ED) and potentially damaging for oral health. Related behaviors, such as binge eating and oral hygiene habits, may equally increase the risk for dental damage. This study aimed to investigate behaviors and habits in patients with ED and SIV in relation to oral health.

**Methods:**

All patients enlisting for treatment in an ED clinic for 1 year were offered to take part in the study. Fifty-four of 65 patients were accepted to participate, and a questionnaire included questions on dietary and oral hygiene habits was included. A subgroup consisting of only those 17 ED patients who reported SIV during the previous six months comprised the sample for this study and received additional questions related to other compensatory behaviors and oral hygiene habits in relation to oral health.

**Results:**

Binge eating before SIV was common (14/17 patients). Time point for SIV after binge eating and the procedures performed after vomiting varied. Tooth brushing after vomiting was common (7/17). Food and drinks during binge eating included mainly items rich in calories (sugar/fat) or acid. All 17 patients believed that vomiting could damage their teeth, but only one of them had informed the dentist about having an ED. A number of oral symptoms were reported. Ten patients considered their oral health to be good/fairly good, while the remaining seven patients reported their oral health as not so good/bad/very bad. Information on how ED could affect their teeth was commonly received from the media.

**Conclusions:**

The dental team should be made aware of the likely detrimental effects of binge eating and vomiting on oral health in patients with eating disorders. The team should also be aware of the cyclical nature of the disease and the similarities and diversities that exist within this group of ED patients. Since ED patients hide their disease from the dental team, this stresses the importance of open and trustful communication between patients and health workers. An organized collaboration between ED clinics and dental professionals is suggested as well as a development of avenues for information about ED and oral health.

## 1. Introduction

Eating disorders (EDs) are psychosomatic disorders and studies have shown that they are often related to oral health problems [1–7]. A recent systematic review of the topic among Western populations reported a lifetime prevalence of EDs to be about 2.6% in females and 0.7% in males [8]. Patients with ED have varyingly shorter or longer periods of different overlapping ED diagnoses and symptoms with different expressivity over time. The overall aim of the behavior of many ED patients is to control their body weight. The most well-known of the ED diagnoses is anorexia nervosa (AN), a diagnosis that is characterized by self-starvation resulting in weight loss, and the disorder can be further divided into the restricting or the binge eating/purging type. Patients with bulimia nervosa (BN), another well-known ED diagnosis, alternate their eating patterns, thus switching between a more regular controlled eating pattern and binge eating (BE). Binge eating is a behavior that includes consumption of an excessive amount of food and drinks, often high in calories, during a specific time period, often two hours. This intake comprises much more food than what is considered normal and a loss of control over both the amount and the choice of the ingested food. To reduce the number of consumed calories, BE is often followed by compensatory behavior, including fasting, vomiting, laxative misuse, or compulsive exercise. Patients with Eating Disorder Not Otherwise Specified (EDNOS) may, according to DSM-IV, present symptoms of AN/BN but which do not completely fulfill the criteria for these diagnoses: such individuals comprised earlier the majority of ED patients. Therefore, the EDNOS category has been criticized for not being specific enough and removed in DSM-V, the most recent DSM-system. Instead, new categories have been added, such as Binge Eating Disorder and Other Specified Eating Disorders [9–11].

Today, according to DSM-V, the severity of AN is based on Body Mass Index (BMI) and is typically below 18.5 for AN diagnosis. In BN, the severity is based on the number of compensatory behaviors per week, such as the use of laxatives, intensive physical training, and/or self-induced vomiting (SIV) [10]. The intention with a compensatory behavior is to reduce the number of calories that have been ingested and thereby achieve weight control and a better emotional feeling. SIV may occur in connection with several ED diagnoses and often take place after an episode of binge eating and consuming an excessive amount of unhealthy food over a longer time period [9, 10].

It is known from earlier studies that both binge eating and vomiting affect oral health negatively regardless of the more specific ED diagnosis and that the choice of food during binge eating is of importance, for example, if the food ingested has cariogenic/erosive potential or not as well as the individual pattern of consumption. SIV will mainly affect the teeth in two ways: (i) indirectly by the cariogenic content of the food ingested prior to vomiting, thus increasing the risk for dental caries; (ii) directly by the acidic content from the stomach reaching the oral cavity during vomiting, which may increase the risk for dental erosion. The number of cariogenic and/or erosive challenges is important for dental health; and also the duration and timing of such events [3, 12]. Tooth sensitivity, which is commonly found among ED patients, may be related to both dental erosion and dental caries [13].

The frequency and level of oral hygiene practices are of importance to prevent dental caries and gingival/periodontal diseases, although tooth brushing after vomiting will increase the risk for tooth wear on often already eroded tooth surfaces [14]. Therefore, it is important for both the patients and the dental team to be aware of such possible negative consequences of oral hygiene practices in relation to acidic challenges such as from SIV. In addition, a more detailed practice of these behaviors and the duration of these events over time, often extending over years, is equally important since the negative impact on oral health is to a large extent cumulative [2–6, 15].

Patients affected by ED are often ashamed about their disease, and it is therefore common that they conceal or even hide their problems from others [11]. This includes not only friends but also the closest family and health personnel, such as medical and dental workers [16]. If significant information regarding an individual's ED is not revealed or ascertained, preventive measures and/or adequate treatment regarding their disease cannot be carried out, and the risk for further oral, psychological, and medical complications are compounded [17, 18]. Therefore, it is important that health workers as a whole are aware of the tendency to conceal their disease by patients suffering from ED to advise and treat these patients correctly.

Studies have shown that ED patients in general, but especially so bulimic patients, have reported concern about their oral health and expressed their need for more specialized knowledge regarding EDs from dentists [19]. At the same time, it has also been shown that Swedish and Norwegian dentists have a limited amount of experience with patients with ED and are reported to have met on average only five and eight ED patients, respectively, during their professional careers [20, 21]. They also reported a lack of clinical experience regarding this group of patients and that their communication abilities needed to address the needs of patients with suspected ED disease are poor.

Knowledge and understanding of the special characteristics and behaviors of patients with ED are critical for assessing their oral health and prescribing adequate dental care. Little is known about the choice of food in relation to BE and the actual procedure/s regarding compensatory behaviors such as SIV in relation to oral health. Therefore, this study aimed to investigate the behavior in ED patients with SIV in relation to binge eating, oral health, and dental care.

## 2. Material and Methods

### 2.1. Selection of Participants

All 65 patients attending the Eating Disorder Clinic, Örebro County Council, Örebro, Sweden over a period of 1 year were invited to participate in the study. Fifty-four of those accepted and an age- and sex-matched control group were selected from a Public Dental Health Clinic in Örebro, Sweden. Results from comparing ED patients and their matched controls were previously reported [3]. Seventeen of the participants in the ED group who reported SIV during the previous six months comprised the sample for the present study.

### 2.2. Questionnaire

A detailed questionnaire was distributed to the participating ED patients (*n* = 54), which comprised questions on dietary and oral hygiene habits and earlier published [4]. Those who reported SIV during the previous six months (*n* = 17) received an additional questionnaire, also including some open-ended questions. The questions were related to compensatory behaviors, such as SIV (onset, frequency, purging, technique for induction, and symptoms afterward), binge eating (time aspects, type of food/drinks, and subsequent vomiting), and oral hygiene habits.

### 2.3. Ethical Considerations

The study was approved by the Ethics Committee in the Örebro Region, Örebro, Sweden (No. 298/03). Informed consent was obtained from all participants and in cases of children also from their parents. An incentive for participation was offered to all control subjects that consisted of a free routine recall for adults and cinema tickets for children.

### 2.4. Statistical Methods

Data were entered into the Statistical Package for the Social Sciences (SPSS version 26.0, IBM SPSS Corp., Armonk, NY, USA). Descriptive statistics was applied, and Wilcoxon's signed-rank test was used for comparison of oral hygiene habits in relatively good and bad disease states.

## 3. Results

Seventeen patients fulfilled the inclusion criteria based on reported vomiting during the previous six months, with a mean age of 23 years (range 16 to 50 years, all women). The mean age of the patient at the onset of ED was 17 years (range 10 to 21 years), and the mean duration of ED was six years (range 1 to 35 years). ED diagnoses according to DSM-IV and retrieved from the specialist ED clinic were EDNOS 58.8% (*n* = 10), BN 35.3% (*n* = 6), and AN 5.9% (*n* = 1). The mean BMI was 21.3 (range 16 to 36).

### 3.1. Awareness and Dental Visits

As regards becoming aware of the existence of having an eating disorder, 12 patients reported that they realized it themselves at first, followed by being told by a relative or a friend (*n* = 5) and parents (*n* = 3). Only one reported that the medical care system had made her aware of having ED and none by a dental care professional. All 17 patients responded that they believed that vomiting could damage their teeth. In response to the question of whether the dentist knew that they had an ED, only one patient had told the dentist while the rest had not given this information, and another one knew that she should have told the dentist but had not done so. Two of them saw “no reason to tell the dentist” about their disease.

Information of how ED could affect their teeth was mainly received from the media (*n* = 7), personnel at the Eating Disorder Clinic (*n* = 4), dentist (*n* = 2), and other sources (*n* = 3: relatives, school nurse etc.), but five patients reported that they had not received any information. Regarding preference for a male or female dentist, all responded that “it did not matter.” Twelve patients reported that they went for regular dental visits only when they had a problem with their teeth (*n* = 3) or did not go to the dentist at all (*n* = 2). The average time since the last dental visit for the 15 patients who reported having visited the dentist was 14 months (range 1 to 48 months).

### 3.2. Binge Eating and Compensatory Behavior

Fourteen of the 17 patients reported “*losing control while eating*” (BE), with an average duration of 39 weeks (range 1 to 260 weeks). Each BE occasion lasted on average for 2.9 hours (range 0.2 to 24 hours). The type of food ingested was mainly sugary items and different soft drinks but also common food and water and/or milk (Table 1).

Eleven of the 14 patients who reported loss of control while eating said that they “always” lose control and two reported doing so “sometimes.” One patient reported to “go to the toilet or do physical exercise” and another reported “panic attack and medication intake” after losing control. The reported mean duration of vomiting behavior was 3.9 years with an average of 8.8 times per week during the previous six months. During periods of feeling relatively bad in their ED, they reported vomiting on average 26.6 times per week (corresponding to 3.8 times per day). The frequency distribution of reported vomiting per week during the previous six months for each patient was 1–3 times (*n* = 8), 4–7 times (*n* = 3), 8–13 times (*n* = 2), and ≥14 times (*n* = 4) (Table 2).

Regarding provocation of vomiting (SIV), the majority (*n* = 11) used fingers down the throat or a combination of “fingers” and “telling the stomach to vomit” (*n* = 3), while the remaining (*n* = 3) reported that they did not do anything as they just “felt sick and vomit.” Nine patients reported that vomiting was induced within 15 min after eating, seven within 30 min, and one patient after more than three hours. The sensations reported after a vomiting occurrence were most often mouth dryness, a general feeling of disgust, and also throat and taste symptoms (Table 3).

### 3.3. Behavior after Vomiting

Fourteen patients reported rinsing the mouth after vomiting: one patient reported only stomach cleaning (explained as drinking after eating and thereafter inducing vomiting to emptying the stomach thoroughly from food and stomach acid by SIV) and another mouth rinsing and stomach cleaning. One patient did not report any rinsing behavior.

Rinsing the mouth with water was the most common action after vomiting (*n* = 16), but also tongue brushing (*n* = 6), eating sugar-free sweets (*n* = 5), drinking cola (*n* = 2), and eating sugared sweets (*n* = 1) were reported (more than one response possible). The volume of drink/liquid used for rinsing was on average 250 mL (range 50 to 1500 mL) after each BE episode. The reasons given for rinsing were to remove the bad taste from the mouth (*n* = 7), clean mouth (*n* = 4), feel better (*n* = 3), remove mouth odor (*n* = 1), remove stomach acid (*n* = 1), and facilitate more vomiting (*n* = 1).

Ten patients reported that they did not brush their teeth immediately after vomiting, while seven patients brushed their teeth, and the reason given for brushing after vomiting was to “get better taste” or “remove odor from mouth” or combinations thereof. In periods of SIV, the patients reported brushing their teeth on average two times per day (range 0–4 times). Commonly available toothpaste brands with fluoride were used, such as Colgate® (*n* = 8) and Pepsodent® (*n* = 3) but also other brands (*n* = 3). Some answered “do not know/no response” (*n* = 3).

There were none or only small nonsignificant differences in brushing practices reported in good or bad disease states regarding mean brushing frequency (2.4 vs. 2.4 times per day), brushing duration (2.9 vs. 3.0 min), or the amount of toothpaste used (1.4 vs. 1.6 cm).

### 3.4. Specific Oral Symptoms

Dental pain or sensitivity while eating or drinking hot or cold items was reported as a daily or weekly occurrence (*n* = 9) and mouth ulcers on a monthly or weekly basis (*n* = 9). Other symptoms were a “lump in the throat” (*n* = 8) ranging in frequency from one to several times per month (*n* = 2), one to several times per week (*n* = 3), or daily (*n* = 3). Recurrent swelling below the ear was reported by three participants.

In response to the question “*do you think that your mouth is dry,*” the reply was yes, every day (*n* = 5), yes, one to several times a week (*n* = 4), and seldom or never (*n* = 8). In response to the question of whether they had noted that their teeth had changed after the onset of their ED, their answers were as follows: more yellowish teeth (*n* = 7); more caries (*n* = 4); more gingival bleeding (*n* = 6). To the question “*do you think your teeth are worn,*” 11 of 17 patients answered in the affirmative: yes, very worn (*n* = 3), yes, fairly worn (*n* = 4), and yes, a little bit worn (*n* = 4). Four of the participants believed that they needed treatment for their worn teeth, ten did not know, and two responded that they did not believe that they needed treatment for worn teeth. Ten patients considered their oral health to be good or fairly good, while the remaining seven patients reported their oral health as not so good (*n* = 2), bad (*n* = 2), or very bad (*n* = 3).

## 4. Discussion

The larger group from which the participants of this study were selected consisted of 54 ED patients and 25 of those reported SIV [3]. In order to reduce the risk of recall bias, only those who reported vomiting during the previous six months were included and only 17 patients out of the 25 fulfilled this criterion. A limitation to this study is the small sample size and that the questionnaire was not validated. Due to the nature of the disease and the accompanying difficulty to get access to ED patients, a higher number of participants could not be achieved. To our knowledge, no validated questionnaire in relation to SIV and oral health exists. The questionnaire in this study was constructed based on what was deemed to be important in relation to oral health. In this regard, the type of food and drinks ingested and the timing for consumption are more important than the amount consumed. Rosen et al. investigated binge eating in 20 women with BN, focusing on type and amount of food related to binge eating during one week with a focus on nutrition and calories [22]. The participants in that study were selected based on an advertisement in a newspaper, while the participants in this study were consecutive patients in a specialist ED clinic for outpatients. Nevertheless, the choice of food consumed in relation to binge eating seems to be quite similar in both investigations, but the timing of food intake in relation to oral health, oral hygiene practices, and other behavior after SIV is only addressed in our study.

In addition to the main questionnaire, a specific questionnaire about vomiting was distributed only to those who reported vomiting and not to the rest of the ED group or the controls. The reason for not including all patients was to avoid a possible risk for initiating such behavior in those who presently did not engage in vomiting or other related activities. The patients in this study were all diagnosed according to DSM-IV by the staff at the ED clinic since this diagnostic system was applied at the time of the study.

Self-induced vomiting is often connected with BN, but only six of the 17 patients selected for this study had a BN diagnosis according to DSM-IV. Most participants (10/17) were diagnosed with EDNOS and only one with AN. This was not surprising since EDNOS patients could, according to the criteria in DSM-IV, practice vomiting. Among AN patients, vomiting is known to be rarer; therefore, it is not surprising that only one of the 17 patients in this study was diagnosed with AN. According to DSM-V, BN is categorized according to the number of inappropriate compensatory behaviors per week, for example, the number of episodes of SIV per week. In this study, six patients had severe or extreme BN (≥8 vomiting episodes per week), while the rest of the patients fulfilled the criteria for mild-to-moderate BN based only on the reported number of vomiting episodes according to DSM-V [10]. Rosen's study of 20 patients with BN reported 199 episodes of BE during one week and the majority of the episodes (93%) were accompanied by vomiting, corresponding to about nine episodes per week [22]. In this study, the average number of SIV episodes was during periods when they did feel particularly bad was much higher (26.6 times), while the reported number of episodes during the last six months was just close to nine per week and in agreement with Rosen's study. Nevertheless, it can be concluded that the frequency of SIV in ED patients varies greatly among patients and over time. Therefore, the health provider needs to retrieve more information about these patients and their behavior to prescribe adequate advice and treatment to minimize the risk for oral health damage.

In general, people may connect low or high BMI with ED patients, especially for a low BMI in AN patients. Most of the patients included in this study had a normal BMI (mean 21.3), reflecting the importance of understanding the variety of physical appearances among different ED patients and that it is often difficult to have a well-grounded suspicion on the presence of possible ED based on body configuration alone [11]. Among Swedish and Norwegian dentists, 56% and 35%, respectively, believed that they could detect an anorectic person outside the dental office, while the corresponding figures for a bulimic person were only 10% and 4% [20, 21]. The first-time awareness of an eating disorder was in 12 of the 17 participants made by the patient and in five patients by parents, relatives, or friends. Only one patient was diagnosed/discovered in the medical system and none in the dental setting or at school. The reason for that could be manifold, but it is obvious that both the dental and medical care systems have not been able to detect the ED patients in this study very effectively, and there is a need to further enhance the possibility for identification of these patients.

All patients in this study knew that vomiting could damage their teeth. Most of the patients went for yearly regular dental visits, and about 40 percent considered their oral health to be impaired (not so good/bad/very bad). Despite this, only one informed the dentist about having an ED. This contrasts with the findings in a Danish study, where proportionately more ED patients informed their dentist about their disease, although about two-thirds of the patients did not inform their dentist about their disorder [19]. Even if the patients in this study were aware of the increased risk for oral complications in relation to their ED, the reluctance to inform their dentist was high. This highlights the special difficulties for the dentist and probably also other caregivers to detect and subsequently provide adequate treatment for ED patients in the clinical setting.

In this study, only two patients reported that they had received information from a dental professional about how their teeth may be affected by an ED disease. This is not surprising since only one of the 17 patients had informed dental professionals about having an ED diagnosis. Without the information provided by the patient and the fact that physical appearance and clinical signs are generally not disease-specific, it is difficult for the clinician to gain a suspicion that they might be presented by an ED patient. Four of the patients in this study reported that they had received information from the personnel at the ED specialist clinic regarding oral health and ED. Healthcare workers in specialized ED clinics have substantial knowledge in the field of ED in general but may have less knowledge on its oral implications [23]. In this regard, a national Swedish survey involving all specialized clinics for eating disorders (*n* = 40), which together treat over 2500 patients annually, was asked by means of a questionnaire to estimate the significance of oral care for patients with ED. Thirty-eight of the 40 clinics responded and 60 percent of the clinics reported that dental problems were frequent in ED patients, 82 percent reported that dental treatment was important in the comprehensive management of ED patients, but only 66 percent gave information on dental care to their patients [24]. However, health workers in specialized ED clinics should be well suited for giving information regarding oral health in relation to ED since patients managed in ED clinics have already revealed their disease and, to some extent, also accepted treatment for this. Therefore, it is likely that they also are more amenable to advise regarding their oral health. As a consequence of the foregoing mentioned study [24], a collaboration between the specialist ED clinic and the specialist dental clinic in Region Örebro was initiated, and all ED patients were offered information and advice on oral health and a dental clinical examination. About 30 percent of the patients accepted this opportunity, while the rest did not [25]. However, many individuals with ED never attend a specialist ED clinic, so advice regarding oral health has to be conveyed in a different manner. In this regard, seven of the patients in this study reported the media as the main source of their knowledge about oral health and ED. Therefore, using social media could be an effective way of informing ED patients that oral health could be affected by their disease, and such a platform would also allow the patients to maintain the privacy (and secrecy) that they prefer.

Binge eating occurred typically prior to SIV and was, as expected, reported by most of the patients (14/17) in this study. The total period of BE reported varied from just one week to periods extending over many years, and the duration of such episodes showed large variation among the patients, lasting from 10 min up to 24 hr. The reported choice of food consumed during BE was mostly rich in calories and cariogenic. The choice of drinks was, to a large extent, water and milk, but also there are other more cariogenic/erosive choices such as light and regular soft drinks and even syrup. Therefore, it is not surprising that some ED patients may also develop oral problems related to dental erosion and dental caries, although the latter is still a question of debate [26].

Most of the patients in this study used the fingers/hand to induce vomiting, although some of them reported inducing vomiting in other ways: for example, “*just felt sick and vomit*” and “*telling the stomach to vomit.*” Inducing vomiting by hand/fingers is a known behavior, but “*just felt sick and vomit*” or “*telling the stomach to vomit*” is not so well reported but could describe the possible association found between BE and gastroesophageal reflux disease and BMI [27, 28]. It is also notable that among those 11 patients who reported SIV using the hand, none of them showed Russell's sign, hardening on the knuckles of the back of the hand due to repeated contact with the upper incisor teeth. This is in contrast with other studies where a prevalence of Russell's sign in BN patients ranged between about 8 and 30 percent [29–32]. It can be speculated that this sign may, in some cases, have been masked or prevented by the patients using softening hand cream.

It is not surprising that periods of intensive SIV would affect the teeth and lead to dental erosive damage, among other problems. It is also very important and necessary that the dentist should be aware of the cyclical nature of such practices among ED patients and the large differences in intensity of risk behavior for oral health.

In this study, several different symptoms were reported to be present after SIV. Some of these are likely to be connected to the large number of acidic challenges from stomach acid during vomiting, for example, bad and sour taste and dryness of the mouth, the feeling of disgust, and having a pain/burning sensation in the throat. Other symptoms are also likely to be connected to the more mechanical irritation of fingers, thus giving rise to the reported “*swallowing difficulties*” and “*paralyzed throat*.” A “*lump in throat*” was reported by about half of the respondents (8/17). This symptom may be attributed to a muscular genesis (medial pterygoid muscle) and the symptom is often seen in patients with temporomandibular disorder or in patients with laryngopharyngeal reflux, but it cannot be excluded that it can be connected to circumstances related to SIV as well [6, 33].

Considering behavior after vomiting, a clear majority reported that they rinse with water, which is in agreement with another study [19]. Some of the participants reported intake of sugary/acidic items like cola or sweet tablets after vomiting, mostly in order to improve taste and odor. Rinsing with water would naturally have a positive effect on oral clearance and elimination of acid from the oral cavity, but using sugary items or a toothbrush might have a negative influence on oral health even though it is clear that tooth brushing is reducing the risk for dental caries. In this study, over 40 percent of the patients reported brushing their teeth with the same purpose as for rinsing to achieve a better taste or odor but brushing after SIV would increase the risk for tooth wear. Tooth brushing after vomiting was considerably higher in this study than the eight percent reported in a previous study [19]. However, it should be noted that the participants in that study had received more information on oral health in relation to their ED than the patients in the present study and thus may have been more informed and motivated to not brush their teeth in following a SIV episode. After completion of our study, all participants received adequate information on oral health measures; in addition, they all agreed to a written letter being sent to their own dentist with advice on how to manage the specific patient's dental situation.

## 5. Conclusion

This study showed that behaviors related to self-induced vomiting and binge eating vary among patients with eating disorders with respect to the duration per episode and over time. Therefore, the consequences for oral health will vary and be affected by the specific compensatory behavior executed in patients suffering from ED. The dental team should be made aware of the likely detrimental effects of binge eating and vomiting on oral health and the large variations of behavior and the cyclical nature of the disease. However, there are also some similarities within this group of ED patients, which needs to be taken into account when prescribing preventive measures. Therefore, it is important that good communication is fostered between patients with ED and health workers and that both parties have a common understanding of how ED may affect oral health. An organized collaboration between ED clinics and dental professionals is needed and the development of specific avenues for information dissemination about ED and its effects on oral health.

## Figures and Tables

**Table 1:** Responses to the open-ended question about intake of food while losing control.

Eating with loss of control	Frequency	Drinking with loss of control	Frequency
Sweets	7	Water	8
Biscuits	7	Milk	4
Pastry	2	Syrup	3
Chips/cheese doodles, snack, nuts	3	Light soft drink	3
Ice cream	2	Regular soft drink	3
Regular food	7	Tea	1
Sandwich	4	Mineral water	1
Carbohydrates/fat/yoghurt	2		
Fruit/salad	2		

Responses from participants to the question “*if you lose control over what you eat and drink*, *what do you eat and drink?*” More than one alternative could be reported (*n* = 17).

**Table 2:** Responses to questions about duration and frequency of self-induced vomiting.

	*n*	Mean	Range	SD
For how many *years* have you provoked self-induced vomiting?	17	3.9	0.3 – 17.0	4.5
How many *times per week* have you been vomiting over the past 6 months?	17	8.8	1.0 – 35.0	10.0
How many *times per day* do you vomit when you feel particularly bad regarding your ED disease?	17	3.8	1.0 – 10.0	2.4

Responses from participants to the question: “*Number of years*, *times per week and times per day of self-induced vomiting*” (*n* = 17).

**Table 3:** Responses to an open-ended question about their feeling in the mouth after self-induced vomiting.

Reported symptom or feeling	No. of responses
Mouth dryness	6
Disgust	6
Throat pain/burning	3
Bad taste	3
Sour taste	2
Other symptoms (swallowing difficulties, throat paralyzed)	2

Responses to the open-ended question “*How do you feel in the mouth after you have been vomiting?*”. More than one sensation could be reported by the patients (*n* = 17).

## Data Availability

The dataset used and/or analyzed during the current study are available from the corresponding author on reasonable request.
